# Evaluation of calcium and magnesium contents in tooth enamel without any pathological changes: in vitro preliminary study

**DOI:** 10.1007/s10266-018-0353-6

**Published:** 2018-03-19

**Authors:** Elzbieta Klimuszko, Karolina Orywal, Teresa Sierpinska, Jarosław Sidun, Maria Golebiewska

**Affiliations:** 1 Department of Prosthetic Dentistry, Medical University of Bialystok, M. Sklodowska-Curie Str. 24a, 15-276 Bialystok, Poland; 2 Department of Biochemical Diagnostics, Medical University of Bialystok, Waszyngton Str. 15A, 15-269 Bialystok, Poland; 3 Department of Materials Science and Biomedical Engineering, Bialystok University of Technology, Wiejska Str. 45, 15-351 Bialystok, Poland

**Keywords:** Calcium, Magnesium, Enamel, Acid biopsy, Atomic absorption spectrometry

## Abstract

Enamel is the structure that covers the entire clinical crown of a tooth. It enables to chew and crush food, and gives a final shape to the crowns of teeth. To evaluate calcium and magnesium contents in tooth enamel and analyse relationships between the study minerals extracted human permanent teeth were cut at every 150 microns and subjected into acid biopsy. The amounts of calcium and magnesium were assessed in the laboratory using atomic absorption spectroscopy with an air/acetylene flame. The lowest calcium and magnesium contents were found on the enamel surface of the teeth. Statistically significant correlation between the calcium and magnesium concentrations was found at a depth between 150 and 900 µm. Calcium and magnesium contents increased with increasing enamel depth. Calcium and magnesium deposits appeared to be stable through all the enamel layers studied. It would be suggested that mineralization/demineralization affect only external layer of the enamel, whereas deeper layers are not affected by these processes.

## Introduction

Enamel is the structure that covers the entire clinical crown of a tooth. A distinctive feature of this tissue is physiological fixity of the structure and composition. It enables to chew and crush food, and gives a final shape to the crowns of teeth [1]. Its thickness varies from 0.01 mm in the cervical margin region to 2.5 mm on top surfaces of crowns [2]. The enamel undergoes wear and tear, and such process affects the majority of population at some period of life. An increasing number of studies suggest that the enamel resistance to external agents depends on its chemical composition and structure which is formed in the process of odontogenesis [3].

The basic structural units of enamel are rods built up of hexagonal hydroxyapatite crystals. Periodic thickened lines on enamel rod that can be seen every 4–8 µm are called cross striation. The distance between those bands may correspond to daily increments of enamel produced [4, 5]. Incremental enamel growth lines are visible in enamel longitudinal cross sections as well. The distance between them is variable and may range from 4–8 µm to 150 µm. The location of those lines shows periodic decrease in enamel matrix secretion during the secretory stage of amelogenesis which occurs physiologically every 5–10 days, and reflects a periodic break in the ameloblast activity [6].

The mature enamel contains inorganic compounds occurring mostly in the form of highly organized and tightly packed crystals that constitute 87% of enamel volume and 95% of its weight [2, 7–9]. The inorganic component of mineralized enamel is composed of 89% calcium hydroxyapatite (Ca_10_ (PO_4_)_6_ (OH)_2_) and small amounts of calcium carbonate (4%), calcium fluoride (2%), and magnesium phosphate (1.5%). Pure hydroxyapatite is composed of 57% phosphorus, 40% calcium and 2% hydroxyl ions. The content of hydroxyapatite crystals in enamel volume is variable and decreases from its surface towards the dentin–enamel boundary. The number, quality and arrangement of hydroxyapatite crystals have impact on appropriate mechanical properties of enamel [10].

Magnesium is present in tooth enamel mainly in the form of magnesium phosphate, and it is an element that has an impact on the quality and anatomy of dental hard tissues [11]. It is essential for the proper development of the tooth structure. Magnesium ions lead to the inhibition of the crystal growth by replacing calcium ions in hydroxyapatite [12]. Magnesium ions play a very import role in the regulation of hydroxyapatite crystal growth as well. They determine physical and chemical stability of crystals as a result of massive loss of organic inhibitors’ molecules between the enamel secretory and maturation stages. This mineral may also affect the alkaline phosphatase activity which catalyses the formation of appropriate hydroxyapatite crystals and may inhibit the transformation of amorphous calcium phosphate to a crystalline form. Magnesium is also a component of the organic matrix of enamel [13].

The aim of the study was to evaluate calcium and magnesium contents in particular layers of tooth enamel and analyse relationships between the study minerals in particular layers of tooth enamel.

## Materials and methods

Fifteen human permanent teeth (central upper incisors) with completed development and without any visible pathological changes were used for the study. The teeth were extracted due to mechanical damage in the area of alveolar process or changes in periodontium. They were obtained from donors between 18 and 21 years of age. They were prepared for the study by strictly adhering to requirements, in accordance with ISO/TS 11405:2015 [14]. The teeth were placed in sterile, securely closing 1.5 mL Safe-Lock tubes, Eppendorf-Netheler-Hinz, Germany. The tubes were marked successively with letters from A to O.

**Fig. 1:**
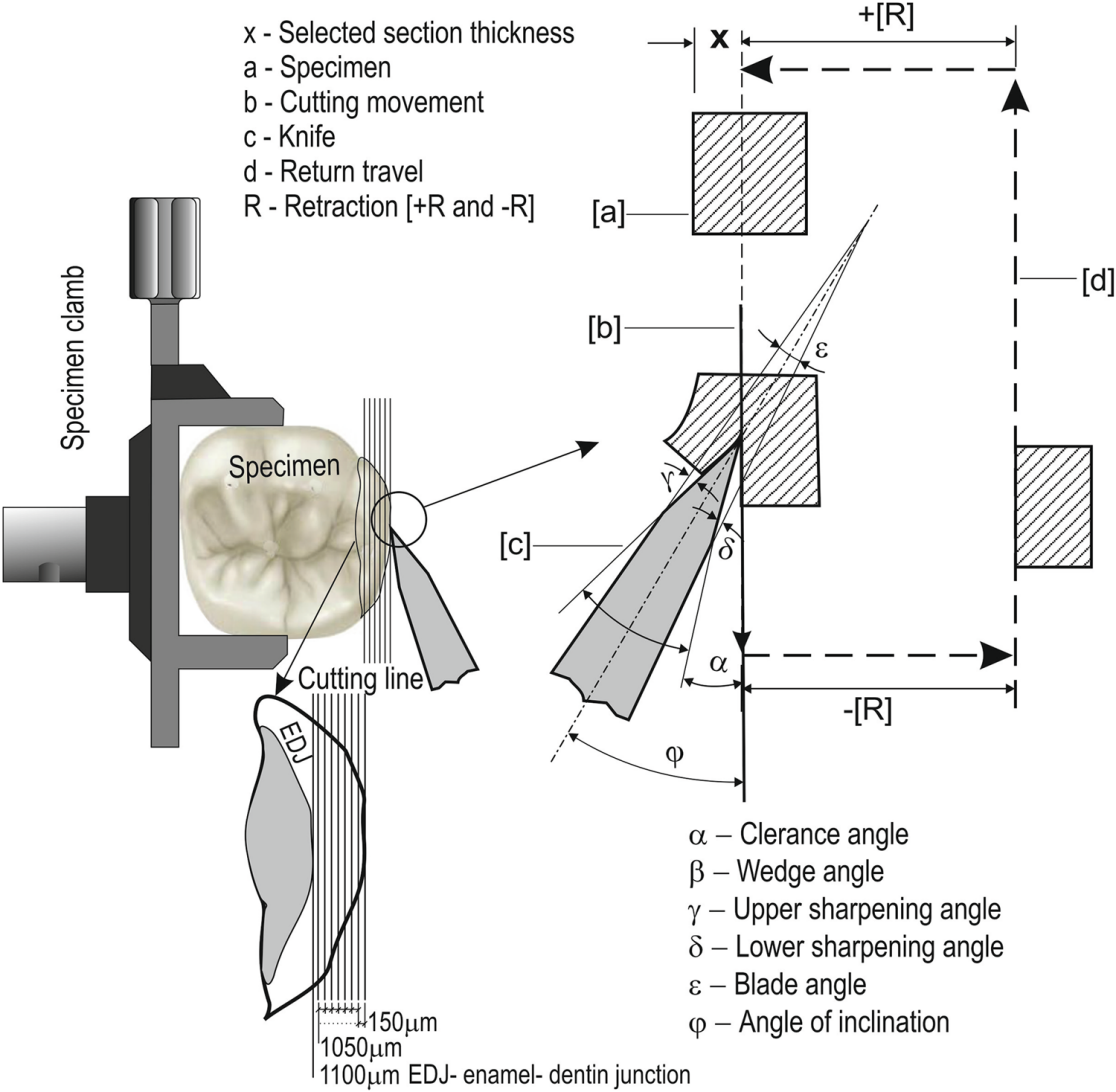
Enamel cutting procedure scheme

Each of the study teeth was placed fixedly in the Microm HM 355 S instrument, International GmbH equipped with a cutting knife made of very hard tungsten carbide WC (Fig. 1). The cutting was started at the point of contact of the knife with the studied tooth Surface. Due to the high hardness of enamel, the dental tissue was cut at the speed of 1 mm/s. The microtome allows cutting with thickness of 0.5 to 150 μm and at the speed of 0 to 430 mm/s. The tooth enamel was cut off every 150 µm alongside the labial surface to obtain the material from seven successive enamel layers for further testing. The central portion of each cut was located in the middle part of the labial surface where the study tooth equator run. The cutting plane and distance between successive cuts were determined on the basis of the location of and distance between the growth lines [15], and the daily amount of deposited enamel was averaged. Then, successive layers were subjected to acid biopsy to determine the calcium and magnesium contents in particular layers.

### Acid biopsy

The first acid biopsy was performed on labial surfaces of the study teeth before they were placed in the Microm HM 355 S instrument, International GmbH and first cuts were made. The material obtained after cutting each enamel layer out was placed on a sterile plate (microscopic cover glass, Menzel, Germany). On the surface of each enamel specimen obtained, 4 Whatman filter paper circles with a diameter of 2 mm and no chemical elements were placed. The circles were saturated with 0.1 mL perchloric acid standard solution (HClO_4_) 0.1 mol/L manufactured by Chempur, Poland. The volume of perchloric acid needed for the study was measured out with a micropipette (Eppendorf Varipette 4710, Eppendorf-Netheler-Hinz, Germany) with a plastic exchangeable tip from the same manufacturer. The enamel specimens were digested for 60 s with acid applied perpendicularly to the study sample, directly on paper circles.

All obtained biopsy specimens were placed in sterile, securely closing plastic 1.5 mL Safe-Lock tubes, Eppendorf-Netheler-Hinz, Germany. Apart from marking with a letter from A to O (designation of the study tooth), each tube was assigned a successive number corresponding to a depth of the study layer: 00—for a specimen collected from the labial surface before the cuts were made, 0—(0 to 150 µm), 1—(150 to 300 µm), 2—(300 to 450 µm), 3—(450 to 600 µm), 4—(600 to 750 µm), 5—(750 to 900 µm), 6—(900 to 1050 µm). The enamel cuts and acid biopsy were performed at the Department of Materials and Biomedical Engineering of the University of Technology.

### Specimens mineralization

The specimens were submitted to the Department of Biochemical Diagnostics. Paper circles were subjected to mineralization for a short period in 1.5 mL concentrated HNO_3_ solution and 0.5 mL ultrapure water. Mineralization is process leading to decomposition and oxidation of organic compounds which are present in the sample and also carrying components to the solution without loss of trace elements. The mineralization was carried out in the Uniclever II microwave mineralizer, Plazmatronika, Poland. The non-diluted mineralizate was used to determine magnesium levels, and the mineralizate was diluted fivefold with ultrapure water for calcium concentration measurements.

### Atomic absorption spectrometry

The calcium and magnesium contents were determined using the Z-5000 polarized Zeeman atomic absorption spectrophotometer, Hitachi, Japan. Concentrations of those elements were determined on the basis of the calibration curve determined by the instrument for a particular element. The calcium and magnesium contents were determined using the flame method with an air–acetylene flame. The concentrations of both elements were determined on the basis of the calibration curve determined by the instrument for a particular element and in working conditions recommended by the instrument manufacturer. All results are the mean value from 3 measurements. The precision of the assay is expressed by relative standard deviation (% RSD), which was < 6% for every tested sample. Both, calcium and magnesium content was determined using the flame method with an air–acetylene flame. Single-element HCL lamp for calcium and magnesium, standard atomizer and fuel flow 2 l/min were used. Working calibration curves for both elements were made by measurements of working standards solutions, prepared by dilution of standards for atomic spectrometry (calcium concentration 1000 mg/L and magnesium concentration 1000 mg/L; Sigma-Aldrich). Concentration of working standards solutions for calcium was 0.0, 0.5, 1.0 and 2.0 mg/L. Concentration of magnesium standards solutions was 0.0, 0.2, 0.4, 0.6 mg/L.

### Statistical analysis

The statistical description of the study characteristics was made. The analysis was performed using nonparametric tests, because the distribution of individual characteristics differed from a normal distribution. The strength of relationships between the pairs of study parameters was measured using the Spearman’s correlation rank coefficient, and its significance was assessed using Student’s t test to evaluate the correlation coefficient. The results for which the calculated probability *p* value was < 0.05 were considered to be statistically significant. Statistica 10.0.PL. computer program was used to make the analysis.

## Results

### Evaluation of calcium (Ca) content in successive enamel layers of the study teeth

The highest mean calcium concentration (14.70333 mg/L) was obtained in a layer 750–900 µm (SD 13.91942; Me 8.75). At the same time, the maximum concentration of this mineral (63.60 mg/L) was found at a depth of 300–450 µm. The lowest mean calcium concentration (1.42 mg/L) was obtained on the enamel surface of the study teeth (SD 0.39406; Me 1.45), where the minimum concentration of this element (0.9 mg/L) was noted as well. The mean calcium concentration for the entire enamel was 10.7175 mg/L.

**Fig. 2:**
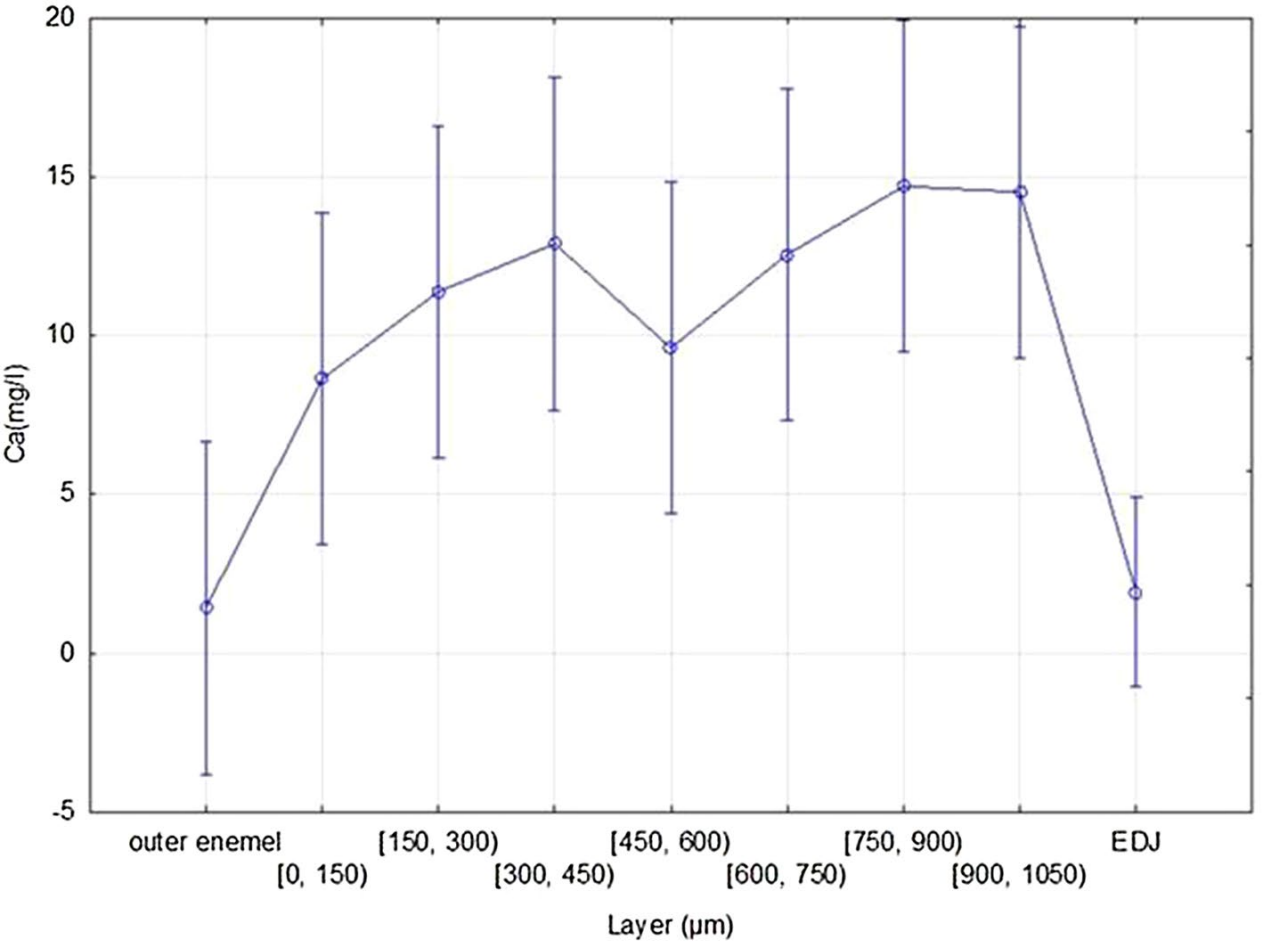
Calcium content distribution in particular enamel layers (00, 0—0 to 150 µm, 1—150 to 300 µm, 2—300 to 450 µm, 3—450 to 600 µm, 4—600 to 750 µm, 5—750 to 900 µm, 6—900 to 1050 µm)

The mean calcium concentrations increase gradually starting from the outer enamel surface to a depth of 450 µm, and then a decrease in concentrations of this element is seen in the 450–600 µm layer (9.61333 mg/L; Me 7.15). Mean concentrations of this mineral start to increase again in subsequent layers up to the maximum value at a depth of 750–900 µm. The calcium content at a depth of 900–1050 µm is slightly lower than maximum mean values (14.52333 mg/L; Me 13.35). A graphical presentation of calcium content distribution at particular enamel depths is shown on Fig. 2.

The relationships between the calcium contents in particular enamel layers taking into consideration the Spearman’s correlation coefficient and the significance level (*p*) for a particular correlation are presented in Table 1. Statistically significant (*p* < 0.05) correlation between the calcium contents was found at various enamel depths.

**Table 1:** Relationships between the calcium contents in particular enamel layers (00—external surface, 0—0 to 150 µm, 1—150 to 300 µm, 2—300 to 450 µm, 3—450 to 600 µm, 4—600 to 750 µm, 5—750 to 900 µm, 6—900 to 1050 µm)

Ca	Spearman’s rank correlation coefficient	*p* significance level
Ca00–Ca3	0.5288	0.043
Ca0–Ca2	0.8225	0.00
Ca1–Ca2	0.6397	0.001
Ca1–Ca3	0.6312	0.012
Ca2–Ca5	0.5555	0.032
Ca3–Ca4	0.6340	0.011
Ca4–Ca6	0.8105	0.00
Ca5–Ca6	0.7038	0.003

### Evaluation of magnesium (Mg) content in successive enamel layers of the study teeth

**Fig. 3:**
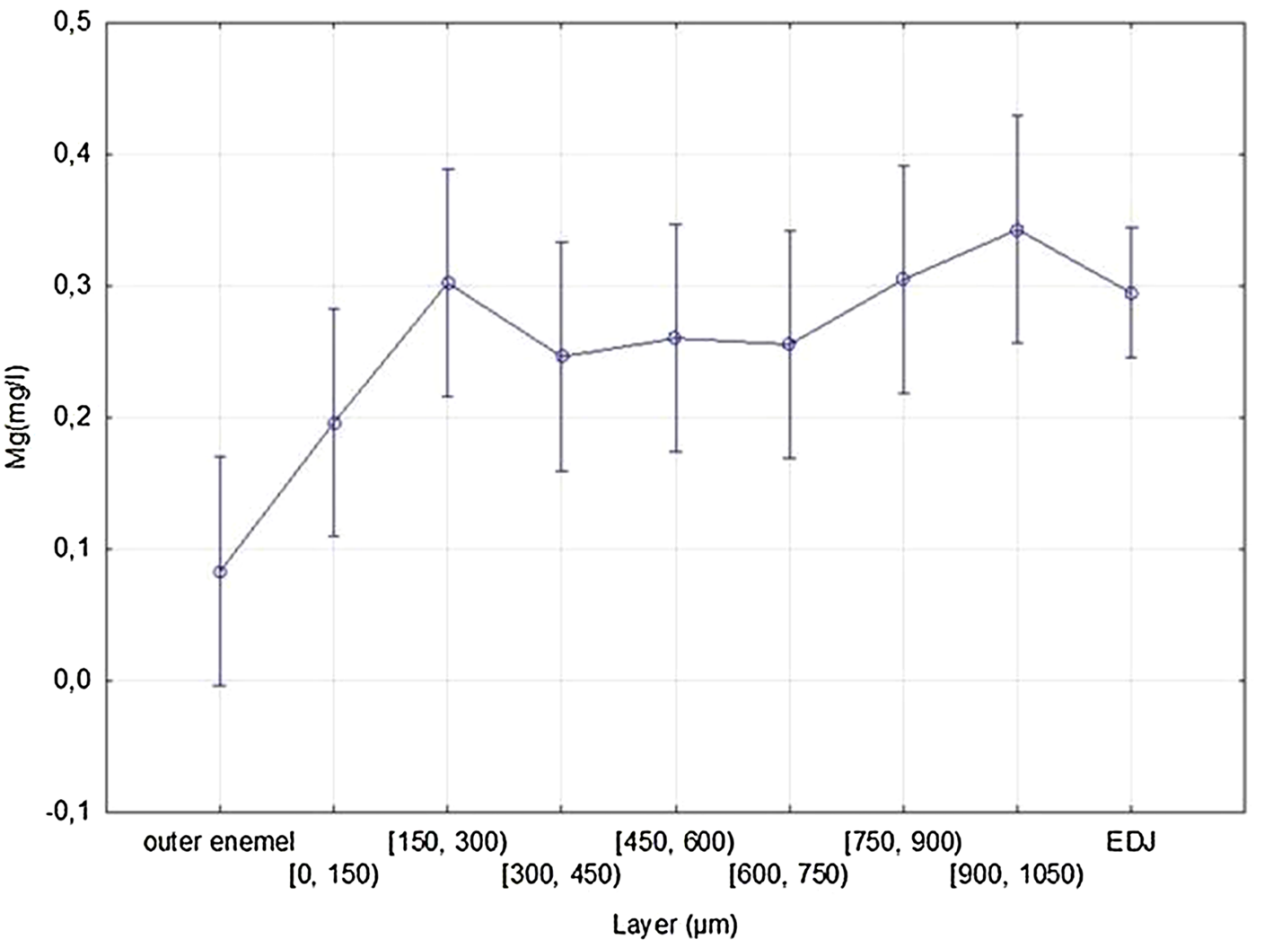
Magnesium content distribution in particular enamel layers (00, 0—0 to 150 µm, 1—150 to 300 µm, 2—300 to 450 µm, 3—450 to 600 µm, 4—600 to 750 µm, 5—750 to 900 µm, 6—900 to 1050 µm)

The highest mean magnesium concentration (0.34267 mg/L) and maximum concentration (1.42 mg/L) were obtained in the 900–1050 µm layer (SD 0.31667; Me 0.23). The lowest mean magnesium concentration (0.08267 mg/L) and the minimum concentration (0.05 mg/L) were noted on the outer enamel surface of the study teeth (SD 0.01792; Me 0.08). The mean magnesium concentration for the entire enamel was 0.2487 mg/L. The mean magnesium concentrations increase gradually starting from the superficial layer of enamel to a depth of 300 µm, and then a decrease in concentrations of this element is seen in the 300–450 µm layer. The concentration remains at the similar level at a depth between 450 and 750 µm, and then the mean concentrations of this mineral increase again up to the maximum values at a depth of 900–1050 µm. A graphical presentation of magnesium content distribution at particular enamel depths is shown in Fig. 3.

The relationships between the magnesium contents in particular enamel layers taking into consideration the Spearman’s correlation coefficient and the significance level (*p*) for a particular correlation are presented in Table 2. Statistically significant (*p* < 0.05) correlation between the magnesium contents was found at various enamel depths.

**Table 2:** Relationships between the magnesium contents in particular enamel layers (1—150 to 300 µm, 2—300 to 450 µm, 3—450 to 600 µm)

Mg	Spearman’s rank correlation coefficient	*p* significance level
Mg1–Mg2	0.5431	0.036
Mg1–Mg3	0.6110	0.016
Mg2–Mg3	0.6849	0.005

### Analysis of relationships between the calcium (Ca) content and magnesium (Mg) content in particular enamel layers of the study teeth

The relationships between the calcium content and magnesium content in particular enamel layers taking into consideration the Spearman’s correlation coefficient and the significance level (*p*) for a particular correlation are presented in Table 3. Statistically significant (*p* < 0.05) correlation between the calcium and magnesium concentrations was found at a depth between 150 and 900 µm.

**Table 3:** Relationships between the calcium content and magnesium content in particular enamel layers (1—150 to 300 µm, 2—300 to 450 µm, 3—450 to 600 µm, 4—600 to 750 µm, 5—750 to 900 µm)

Ca/Mg	Spearman’s rank correlation coefficient	*p* significance level
Ca1–Mg1	0.8702	0.00
Ca2–Mg2	0.9286	0.00
Ca3–Mg3	0.6547	0.008
Ca4–Mg4	0.9042	0.00
Ca5–Mg5	0.8870	0.00

## Discussion

Information about metabolic and physiological processes occurring during the ontogenetic development is contained in enamel [16]. Minerals contained in the inorganic portion of a tooth should occur in strictly defined concentrations, as they determine its hardness, resistance to environmental influence and appropriate direction of biochemical transformations, among others. The absence of some minerals in the tooth tissue may affect the content of other minerals and result in a greater tooth vulnerability to dental caries and other pathological agents [17]. Therefore, the studies to evaluate the calcium and magnesium contents at various enamel depths seem to be fully justified and are important to determine the enamel resistance to harmful agents. A decision was taken to evaluate the content of some minerals (calcium and magnesium) at various enamel depths as part of the paper, since these are the main enamel-building minerals. The objectives of the paper cannot be accomplished in the clinical setting, because the method would be too invasive, leading to complete destruction of the tooth structure. Therefore, human teeth free from dental caries and other pathological processes, and stored in conditions allowing for the evaluation and analysis of enamel mineral composition were used for in vitro studies [14]. However, it should be taken into consideration that the content of a particular mineral in a biopsy specimen may indicate a low enamel vulnerability to dissolution under the influence of acid applied, or it may indicate the actual mineral content in enamel [18].

According to the available literature data, the mean calcium content in the superficial layer of tooth enamel free from pathological changes is 1.85 mg/L [3]. In this paper, the mean calcium concentration of 1.42 mg/L was obtained on the enamel surface of the study teeth, while the mean concentration of this mineral for the entire enamel was 10.7175 mg/L. It has been found that the calcium content increases with enamel depth. According to some authors, the content of calcium and phosphates decreases starting from the surface to enamel amelodental unit, which is correlated with the decreasing mineral density of enamel [19]. The studies carried out by Pawlus et al. [20] in the group of 20-year-old subjects have shown the content of calcium and magnesium in teeth of 307.77 and 7.10 g/kg, respectively. Other literature data show different results. Some scientists say that calcium is one of the most important chemical determinants of enamel quality [21]. Its concentration in healthy enamel tissue decreases from the enamel surface towards the dentin, and varies between 36.5 and 40.0% by weight.

There are no literature data on relationships between the calcium contents at various enamel depths. The tests described in the paper have shown that an increase in the calcium content in one enamel layer affects its content increase in another one. Based on this it may be assumed that the mineralization process of superficial enamel layers influences the course of this process and the final mineral composition of layers that undergo mineralization at a later point in time. However, it is also necessary to emphasize that external layer of the enamel is affected by the environment of human mouth that may influence the content of calcium in this layer.

The studies available in the literature suggest that the mean magnesium concentration in enamel ranges between 0.25 and 0.56% (w/w) and may be up to three times higher in inner layers compared to the enamel surface without any pathological changes, where the mean magnesium concentration of 0.33 mg/L was noted. According to Sendur [22], the magnesium concentration in enamel and dentin of healthy teeth is higher compared to teeth with dental caries, and is around 6.1 g/kg. Some studies suggest that the number of magnesium ions in enamel was decreasing with biopsy depth, and constituted around 0.81% of its inorganic substances. In the same studies, the mean concentrations of magnesium ions of 10.84 g/kg on the enamel surface, and of 6.05 g/kg in the subsurface enamel layer were noted [23]. Other reports demonstrated that magnesium content in the outer, the most superficial enamel layer, was greater compared to deeper enamel layers (the magnesium contents of 133.12 mmol/kg on the enamel surface, and of only 67.57 mmol/kg in the deepest layer were found). The same study enabled to determine the mean magnesium content in the entire enamel (78.05 mmol/kg) [11]. Some *scientists* say that ion exchange at the enamel–saliva and enamel–plaque interfaces and a certain accumulation of this element with age may account for the greater magnesium content in outer enamel layer [11, 22, 24]. There are reports that the magnesium content decreases with the progress of the mineralization process, and that is why its amount decreases with distance from the dentino–enamel junction to the tooth surface [22]. Such hypothesis is confirmed by the results obtained in this paper. It has been demonstrated that the magnesium content increases with enamel depth (a slight decrease compared to the preceding layer was noted at a depth of 300–750 µm). According to the tests presented in the paper, the mean magnesium concentration in the entire enamel was 0.2487 mg/L.

The experiments by Aoba et al. [13] have shown that an increase in calcium transport during the enamel maturation stage results in a direct increase in forces responsible for precipitation and accelerates the replacement of magnesium ions adsorbed on the surface of crystals, leading to a lower inhibitory effect on the growth of enamel crystals [11, 13]. Other studies have shown that in acid environment calcium content in hydroxyapatite crystals is negatively affected by the high magnesium content [17, 25]. Magnesium ions act as a mineral growth inhibitor in solutions containing calcium and phosphorus [26, 27]. This would indicate that the magnesium content decreases with growing calcium content, but the test results obtained as part of the paper do not confirm it. The conducted enamel analysis has shown that the magnesium content increases with growing calcium content at a depth between 150 and 900 µm. It means that the content of one of those minerals directly influences the content of the other one. Those relationships do not apply to the superficial layer, which may be explained by the exchange of ions with the components in saliva and the layer at the dentin interface. Therefore, it may be concluded that the content of those two minerals was established during the enamel development stage and is related to the location of the lines of Retzius and phased maturation of enamel [28].

The studies on teeth with hypomineralization showed that the mean calcium-to-phosphorus ratio was significantly lower, and the magnesium content was slightly higher compared to healthy teeth [29]. Since calcium and magnesium are interdependent, and the calcium content should increase with growing magnesium content, a suggestion arises that the reduction in calcium-to-phosphorus ratio in teeth with abnormal enamel structure is related to an increase in the phosphorus content, and not the elimination of calcium.

Changes in the chemical composition of teeth may be age-dependent [20]. However, the studies carried out by Tsuboi et al. showed no increase in the calcium and magnesium contents with age, both in permanent and primary teeth [30]. This may indicate that the accumulation of those elements occurs when tooth buds are being formed, even before the eruption of teeth. The delivery of those minerals to the body at a later point in time has no impact on the general pool of magnesium and calcium in teeth. This assumption is confirmed by the results from studies on tooth buds, which have demonstrated that the calcium and magnesium contents increase with the age of foetuses. This shows that the most intensive mineral composition transformation process occurs in foetal life. Therefore, magnesium and calcium are elements of which the content remains at relatively stable levels after a tooth is formed (no differences between the contents of those minerals in tooth buds and permanent teeth) [20].

## Conclusion

The lowest calcium and magnesium contents were found on the enamel surface of the study teeth. The calcium and magnesium contents increased with increasing enamel depth. The calcium and magnesium contents in outer enamel layers affect an increase in the content of unipolar minerals at deeper enamel layers.
